# The Role of miRNA in Endometriosis-Related Infertility—An Update

**DOI:** 10.3390/ijms26125862

**Published:** 2025-06-19

**Authors:** Hanna Surmann, Ludwig Kiesel

**Affiliations:** Department of Gynecology and Obstetrics, University of Muenster, Albert-Schweitzer Campus 1, 48149 Münster, Germany; hannaelisabeth.surmann@ukmuenster.de

**Keywords:** endometriosis, infertility, miRNA, diagnostic, therapy

## Abstract

Endometriosis, affecting up to 10% of women in their reproductive years, is a chronic and multifactorial disease characterized by the presence of endometrial tissue outside the uterus. Traditionally associated with pain and infertility, recent studies highlight its systemic nature, implicating inflammatory, immunological, and hormonal dysregulation in its pathogenesis. This paper explores the emerging role of microRNAs (miRNAs) in the pathophysiology of endometriosis and its related infertility. Evidence suggests that dysregulation of specific miRNAs influences cellular proliferation, migration, and progesterone resistance, thereby contributing to the development and progression of endometriotic lesions. Additionally, altered miRNA expression profiles hold promise as non-invasive biomarkers for improving diagnostic accuracy and as potential targets for novel therapeutic interventions. Although current diagnostic methods, such as laparoscopy, remain the gold standard, the integration of miRNA-based approaches could reduce reliance on invasive procedures and enhance treatment outcomes. Ultimately, further research—particularly regarding the interplay between endometriosis and infertility—is crucial to fully elucidate these complex mechanisms and foster the development of more effective diagnostic and therapeutic strategies.

## 1. Introduction

Endometriosis is a chronic disease which affects up to 10% of women during their reproductive years, impacting approximately 190 million women globally. It is defined by the presence of endometrium tissue outside of the uterus. The disease has been linked to a variety of symptoms, including chronic pelvic pain, dysmenorrhea, dyschezia, cyclic bloating, hematuria, dysuria, dyspareunia, and infertility [1]. Apart from these classic symptoms, endometriosis has an impact on the inflammatory, immunological, and hormonal environment in the bodies of affected patients [2]. The prevalence of comorbidities, including rheumatoid arthritis, migraine, cardiovascular disease, and other diseases, is notably increased in patients diagnosed with endometriosis [3,4,5]. Thus, it should also be recognized as a systemic disease. The disease burden of endometriosis manifests in a variety of ways, extending beyond the physical symptoms to significantly impair quality of life, mental health, and socio-economic stability. A significant proportion of patients encounter delays in diagnosis that can extend up to seven to ten years [6]. This delay frequently results in more severe disease progression and contributes to comorbidities and infertility.

Infertility is a disease that is defined as the non-occurrence of pregnancy after 12 months of regular sexual intercourse without contraception. It is estimated that approximately 8–12% of all couples are affected by infertility. There are a number of potential etiologies associated with female infertility. Among these etiologies are sexually transmitted diseases, such as chlamydia and gonorrhea; Turner syndrome; congenital anomalies; polycystic ovarian syndrome; endometriosis; and other conditions [7]. It is also important to note either the male or the female can be the cause of the infertility of the couple. In some cases, both partners contribute to the condition [8].

Endometriosis is one of the most common causes for infertility. It may be related to blocked fallopian tubes due to endometriosis lesions, diminished ovarian reserve due to endometriomas or previous surgeries, an altered hormonal environment, or inflammatory changes in the endometriosis patients. Endometriosis is a heterogenic disease. Thus, all possible mechanisms responsible for infertility may not yet have been established. About 30% of endometriosis patients also suffer from infertility, whereas about half of the patients that are diagnosed with infertility can also be diagnosed with endometriosis [9]. Recent findings indicate that in instances where anatomical distortions are minimal, molecular and immunological alterations in the endometrium may impede implantation, thereby exacerbating the complexity of fertility outcomes in these patients [10].

Assisted reproductive technologies, particularly in vitro fertilization, have demonstrated efficacy in enhancing pregnancy outcomes in endometriosis-associated infertility. However, the success rates of these treatments may vary depending on the severity of the disease and the age of the patient.

Especially since there are many women who are affected by endometriosis and its associated infertility, it is important to know the connections between the two diseases in order to be able to offer the best possible treatment. To this end, the individual factors of each patient should always be considered. The field of medicine has recently witnessed a paradigm shift towards personalized medicine, with the integration of genetic, hormonal, and environmental profiles being a key area of research. This approach aims to personalize treatment regimens for patients suffering from endometriosis-related infertility, with the dual objectives of enhancing symptom management and preserving fertility.

## 2. miRNA and the Pathophysiology of Endometriosis

The pathogenesis of endometriosis has not yet been fully understood. Endometriosis is a heterogenic disease, and several different theories regarding its pathogenesis have developed over the years. One of the first theories includes retrograde menstruation, which could explain the presence of endometriotic lesions in the peritoneum. It does not explain the occurrence of extragenital endometriosis or any systemic effects of the disease. Moreover, the theory fails to elucidate that retrograde menstruation, which occurs in 90% of women, only leads to a prevalence of endometriosis in 10–15% of women. Consequently, retrograde menstruation may play a role in the pathogenesis of the disease, but alone, it cannot fully account for disease development. Other theories of pathogenesis include the hematogenous and lymphovascular dissemination theory or the stem cell theory. Recent research additionally proposes epigenetic and genetic etiology as a cause for endometriosis [9].

The alteration of miRNA in patients with endometriosis is one factor in the epigenetic theory of endometriosis pathophysiology. miRNAs are a type of short non-coding RNAs, consisting of 18–22 nucleotides, that can be found ubiquitously in the body. miRNAs are transcribed from the deoxyribonucleic acid (DNA) but not translated into proteins. The function of miRNAs is the regulation of the Ribonucleic acid (RNA) communication network to control proteins and cellular functions. This process is known as the post-transcriptional regulation of gene expression. miRNAs have been shown to repress the translation of different messenger RNAs into proteins. Another function of miRNAs is their role as chemical messengers. In contrast to the regulation of gene expression, which predominantly occurs in the cytoplasm, the chemical messenger function can be distributed throughout the entire organism. miRNAs have been found to be present in extracellular vesicles within a variety of bodily fluids, including blood, urine, and peritoneal fluid [11]. The ability of miRNAs to modulate a variety of biological processes, including inflammation, proliferation, angiogenesis, tissue remodeling, and disease progression, underscores their critical role in biological systems. This discovery has led to the identification of an association between miRNAs and a variety of diseases, including cancer and endometriosis [12].

It has been suggested that the alteration of miRNA plays an important role in endometriosis pathophysiology [13]. It has been found that the alteration of miRNAs has an effect on endometriotic cell proliferation, endometriotic cell migration, progesterone resistance, and inflammation. All of these factors are crucial for endometriosis pathogenesis and lead to the common symptoms of endometriosis patients. A significant number of miRNAs have been observed to exhibit differential expression in women diagnosed with endometriosis. This observation has led to the hypothesis that these variations may contribute to the pathogenesis and progression of the disease.

miR-370-3p is downregulated in women with endometriosis, leading to an increase in SF-1 mRNA, which regulates estrogen biosynthesis. miR142-3p is another miRNA which is downregulated in endometriosis patients, resulting in an increase in cell proliferation and metastasis in ectopic endometriosis lesions. It has been shown that an injection of miRNA-142-3p causes a shrinkage of these lesions [13]. miRNAs from the miR-200 family are responsible for cell migration in endometriosis, which could explain the fact that endometriosis lesions might develop in almost every part of the human body [13].

One of the reasons for infertility in endometriosis patients might be implantation failure due to progesterone resistance. Brichant et al. [13] showed that miR-29c and miR-194-3p play a role in the resistance to progesterone. The alteration of other miRNAs was shown by several investigators. let-7b and miR-135a are decreased in endometriosis patients and might also be possible targets for new therapeutical options of the disease [14]. Seifer et al. [15] was able to detect a decrease in let-7a when assessing circulating miRNAs in murine experimental endometriosis. miR-451a is a miRNA that has been observed to be significantly elevated in the serum of women with endometriosis. In the future, it may be used as a mode of diagnosis, in combination with the alteration of other altered miRNAs [16].

In conclusion, there are many increased or decreased miRNAs observed in women with endometriosis which might be a part of the pathophysiology and characteristics of the disease.

## 3. miRNA and Infertility

miRNAs have been identified as playing a crucial role in infertility, particularly in cases of endometriosis-associated reproductive challenges. Studies have identified miR-126 and miR-145 as significantly upregulated during the midluteal phase in infertile women with endometriosis, suggesting their potential as prognostic markers and connection to infertility. Furthermore, the levels of these miRNAs were significantly elevated in non-pregnant patients with endometriosis in comparison to pregnant patients the disease after undergoing assisted reproduction treatment. This supports their relationship with endometriosis-associated infertility [17]. miR-126 has been shown to play a role in angiogenesis and vascular remodeling. miR-145 is known to inhibit proliferation and invasion, which may impair implantation processes in endometriosis patients [17].

A large-scale screening of 2651 miRNAs revealed 34 key miRNAs linked to endometriosis-related infertility, with miR-6818-5p, miR-498, miR-1910-3p, miR-501-5p, and miR-31 being of particular note [18]. miR-6818-5p has been identified as a key player in the complex interplay between the sperm and the oocyte. This results in the modulation of the Hepatocyte nuclear factor 4 alpha (HNF4A) gene expression, which plays a role in the AMP-activated protein kinase (AMPK) signaling pathway and the calcium signaling pathway. It has been established that both of these pathways are essential for oocyte maturation and the interaction between sperm and oocyte that results in fertilization [19]. miR-498-3p has been identified as an inhibitor of histone deacetylase 4 (HDAC4), a critical regulator of decidualization in endometrial stromal cells, a process necessary for successful implantation. The knockdown of HDAC4 has been shown to induce autophagy and apoptosis in placental trophoblast cells, processes that can compromise placental development and function. These observations suggest that miR-498-3p may contribute to endometriosis-associated infertility by disrupting decidualization and promoting trophoblast cell dysfunction through the downregulation of HDAC4 [20]. To date, there is limited research on the role of miR-1910-3p, although it has been implicated in the pathogenesis of gynecological cancers such as breast cancer and endometrial carcinoma [21,22]. Its involvement in endometriosis has not yet been thoroughly investigated. However, emerging evidence suggests that miR-1910-3p may influence infertility, as studies in patients with polycystic ovary syndrome have demonstrated its role in regulating endometrial receptivity through modulation of Integrin beta-3 subunit (ITGB3) and Homeobox A10 (HOXA10) expression. These findings raise the possibility that miR-1910-3p may exert similar effects in the context of endometriosis-associated infertility [23]. No research has been published on the connection between miR-501-5p and endometriosis or infertility. Its possible influence, which has been suggested via large-scale screening by Dabi et al. [18], is so far unknown. Further research is required to elucidate the specific pathways involved. miR-31 has been associated with recurrent implantation failure in patients undergoing assisted reproductive treatments, suggesting its potential role in impaired endometrial receptivity. It is notably upregulated during the proliferative phase of the endometrial cycle, although the precise molecular pathways through which it exerts its effects remain unclear. Despite the lack of studies specifically focused on patients with endometriosis, the observed patterns suggest that miR-31 may similarly contribute to infertility in this population. Its consistent expression profile highlights its potential as a biomarker for assessing endometrial receptivity, warranting further investigation into its mechanistic role and clinical relevance in endometriosis-associated infertility [24].

Recent studies have demonstrated the potential of miRNA levels, specifically those of miR-100-5p and miR-21-5p, to serve as biomarkers for anti-Müllerian hormone levels and the assessment of ovarian reserve status [25]. miR-100-5p has been shown to inhibit the proliferation of endometrial stromal cells and induce apoptosis in vitro, indicating a potential role in the regulation of endometrial function; one target gene is Homeobox A1 (HOXA1), which is involved in uterine development. In a mouse model, the overexpression of miR-100-5p significantly reduced embryo implantation rates by impairing uterine receptivity, suggesting its contribution to implantation failure [26].

miRNAs have been identified as playing a crucial role in fetal–maternal communication and influencing endometrial receptivity, which is vital for implantation [27]. Generally, miRNAs modulate immune tolerance, angiogenesis, and cell adhesion, making them key regulators of endometrial receptivity.

The let-7 family of miRNAs also plays an important role in regulating endometrial receptivity, particularly through its interaction with insulin-like growth factor 1 (IGF1), a key player in cell growth and differentiation processes. In addition, let-7 is essential for various processes related to cell differentiation, and its downregulation has been observed in both mouse models of endometriosis and in human studies. Interestingly, treatment with aromatase inhibitors has been shown to increase let-7 levels, suggesting a potential therapeutic avenue for restoring normal endometrial function. However, the full range of let-7 targets remains incompletely characterized [28].

The dysregulation of miR-543 (downregulated) and miR-135a/b (upregulated) in endometriotic tissue during the implantation window indicates impaired endometrial receptivity [27,29]. miR-29c upregulation affects FK506-binding protein 4 (FKBP4), contributing to progesterone resistance and negatively impacting implantation [27].

miR-22-5p is downregulated in women with endometriosis, leading to increased Tet methylcytosine dioxygenase 2 (TET2) activity and altered methylation of genes like the progesterone receptor, HOXA10, and Estrogen Receptor 2 (ESR2), which contributes to progesterone resistance and implantation failure [30].

**Figure 1:**
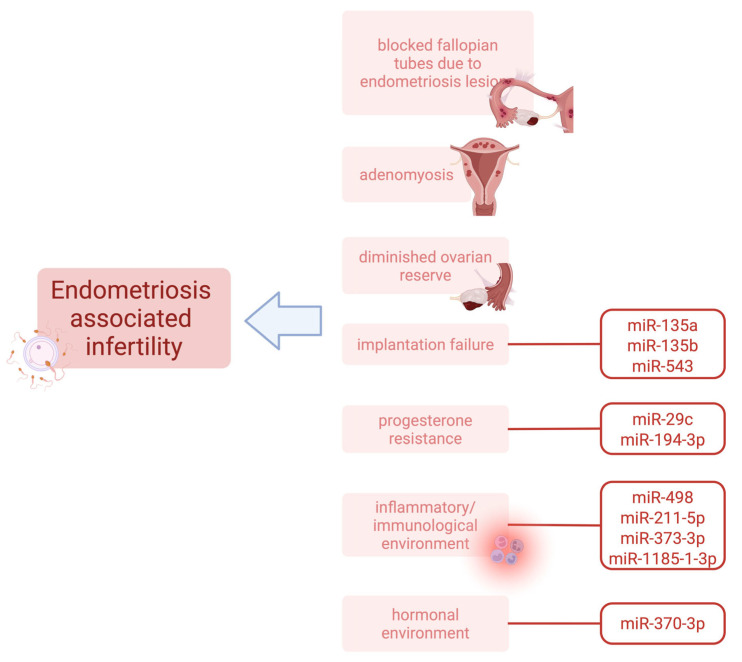
Factors of endometriosis-associated infertility (created in BioRender; Surmann, H. (2025) https://BioRender.com/n22xkdd, accessed on 13 June 2025).

These findings underscore the substantial impact of miRNAs in infertility and their potential as diagnostic and therapeutic targets (Figure 1). The functions of all miRNAs identified in relation to endometriosis-associated infertility are not currently known. The known functions of miRNAs in this context are summarized in Table 1.

## 4. miRNA as a Diagnostic Tool for Endometriosis and Associated Infertility

Currently, laparoscopy remains the gold standard for diagnosing endometriosis, as the accuracy of sonography alone is insufficient. However, transvaginal sonography or MRI can often provide a well-founded suspicion of endometriosis. Despite this, histological confirmation via laparoscopy is often still required [1]. Early and accurate diagnosis is crucial for managing endometriosis and preventing progression to infertility.

Surgical excision of endometriomas has been shown to negatively impact ovarian reserve, as evidenced by a significant reduction in anti-Müllerian hormone levels post-surgery [32]. Additionally, endometriomas themselves can cause ovarian reserve damage [33]. Since antral follicle count is also significantly reduced after endometrioma surgery [33], surgical interventions should be approached cautiously, particularly in younger patients considering future pregnancies. To avoid unnecessary surgery, alternative diagnostic approaches are needed.

Potential biomarker-based methods, such as miRNA analysis from blood, saliva, urine, or endometrial tissue, are being explored but are not yet included in diagnostic guidelines [34]. Urinary biomarkers have been investigated but have not yet yielded reliable diagnostic results due to small patient cohorts and heterogeneous findings [35].

In contrast to those findings, blood-based miRNA biomarkers show promising results. A total of 63 miRNAs with higher expression in endometriosis patients have been identified, although only 14 have been validated in multiple studies, highlighting the need for further research [34]. One study identified six promising miRNAs—miR-125b, miR-150, miR-342, miR-451a, miR-3613, and let-7b—showing altered expression in endometriosis patients. Their combined diagnostic performance demonstrated an area under the curve (AUC) of 0.939, and clinical trials are ongoing to develop a potential diagnostic test [36]. Notably, no influence of hormonal contraception on miRNA expression was found in this study, suggesting that a future diagnostic test could remain independent of contraceptive use.

Furthermore, a saliva-based test for endometriosis is already available. It utilizes a combination of 109 miRNAs and has shown a high diagnostic accuracy, with an AUC of 0.983 [37]. However, the study cohort was limited to 200 patients, exclusively including those experiencing pelvic pain and excluding individuals with infertility as their sole symptom. Therefore, further studies with larger and more diverse patient populations are required to validate its broader applicability.

A number of miRNAs have been demonstrated to exhibit alterations in endometriosis and infertility, as well as in endometriosis-associated infertility. As previously indicated, the development of diagnostic tests for endometriosis that are based on miRNA is a primary focus within the research community, and such testing has been previously proposed. To date, there is an absence of specific diagnostic tests for endometriosis-associated infertility. This approach would be a worthwhile consideration for future implementation. The development of a diagnostic test based on miRNAs is a plausible prospect, given the substantial involvement of these molecules in the associated mechanisms. Therefore, it is crucial to identify the key miRNAs involved in endometriosis-associated infertility.

While miRNA-based diagnostics are not yet part of standard clinical practice, ongoing research indicates promising potential for the use of non-invasive diagnostic tools.

## 5. miRNA as a Therapeutic Option

Current treatment options for endometriosis include the surgical resection of lesions and hormonal therapies, with the latter being unsuitable for women seeking to conceive. A more desirable approach would consist of targeted and personalized therapy. RNA therapy has demonstrated significant potential in the treatment of various oncological diseases, with phase 2 clinical trials underway for conditions such as lymphomas, leukemia, hepatitis C, mesothelioma, and Crohn’s disease [38]. At present, no therapy based on miRNAs is available for endometriosis. However, there are ongoing clinical trials investigating several RNA-based therapies, including those targeting endometriosis [38].

A potential avenue for therapeutic intervention involves the use of mimics to restore downregulated miRNAs or inhibitors to counteract overexpressed miRNAs. However, the potential for adverse effects remains to be evaluated. Most clinical trials currently testing miRNA-based therapeutic approaches for different diseases only include inhibitors. Few clinical trials are currently performed using miRNA mimics.

The development of miRNA-based therapies for endometriosis and infertility faces several important challenges that must be carefully addressed. When considering the use of miRNA inhibitors, it is essential to perform thorough loss-of-function studies both in vitro and in vivo to better understand the specific roles of targeted miRNAs in disease pathology. Similarly, gain-of-function studies are critical when evaluating the potential of miRNA mimics, as they help elucidate the effects of increased miRNA activity within the cellular environment. However, one significant obstacle is the inherent instability of miRNAs in vivo, primarily due to degradation by nucleases, which can limit their therapeutic efficacy. Therefore, it is crucial to consider the method of delivery, particularly the choice between systemic and local administration, depending on the targeted tissue and the desired therapeutic outcome. To enhance stability and facilitate efficient cellular uptake, miRNAs can be encapsulated in lipid-based nanoparticles, a strategy that has shown promise in promoting delivery to target cells.Alternatively, viral vectors offer another effective method for transferring miRNA constructs into cells, although issues related to immunogenicity and insertional mutagenesis must be carefully evaluated.

Another promising approach involves the use of miRNA sponges, which are engineered molecules containing multiple target sites that can bind and downregulate one or several miRNAs simultaneously. While miRNA sponges offer the advantage of targeting entire miRNA families or related pathways, it is critical to always consider the possibility of off-target effects, which could lead to unintended consequences and impact other important cellular processes [39]. Overall, the successful translation of miRNA therapies into clinical practice will require meticulous optimization of these delivery systems, careful evaluation of potential side effects, and rigorous preclinical validation.

A notable study by Li et al. [40] reported that treatment with a miR-451a inhibitor resulted in a substantial reduction of endometriotic lesions in a murine model. This inhibition also influenced the gene expression of pivotal factors known to be involved in the pathophysiology of endometriosis. Concurrently, Sahin et al. [41] induced endometriosis in mice and administered let-7b-5p via intraperitoneal injection, resulting in a substantial reduction in endometriotic lesions compared to the results for the untreated controls. This approach also elicited notable changes in the expression of genes associated with endometriosis pathophysiology.

Importantly, all investigations of miRNA-based therapeutic strategies for endometriosis remain at the preclinical stage, with no interventions having progressed to human trials. Published studies have validated target engagement and mechanistic effects in vitro, using primary endometrial stromal cells or established cell lines, as well as in vivo, using surgically induced murine models. Until rigorous pharmacokinetic, toxicology, and dose-escalation studies are completed, miRNA-based therapies for endometriosis-related infertility will remain experimental and not yet ready for clinical application.

Collectively, these experimental findings underscore the potential of miRNA-based therapy to yield promising results in preclinical and animal models, providing a foundation for future treatment strategies. Nevertheless, before such therapeutic interventions can be applied in clinical practice, further research is necessary, especially in the form of clinical trials, to evaluate their safety and efficacy.

## 6. miRNA as a Therapeutic Option or Addition to Assisted Reproduction Techniques—Future Possibilities

miRNA-based therapy has emerged as a promising avenue for enhancing fertility outcomes in patients with endometriosis, particularly by targeting progesterone resistance. A recent study by Tang et al. [42] has identified specific miRNAs, including miR-21-5p, miR-194-3p, miR-297, miR-169a, and miR-92a, that play a crucial role in regulating progesterone resistance. Furthermore, the utilization of miRNA mimics or inhibitors has been investigated as a potential therapeutic approach for restoring normal endometrial function and counteracting the effects of dysregulated miRNA expression in patients with endometriosis [43].

Early diagnosis using non-invasive methods based on miRNAs could facilitate timely intervention, potentially reducing the severity of endometriosis. This, in turn, may lead to a lower incidence of blocked fallopian tubes, fewer endometriomas, and less impact on the ovarian reserve, ultimately decreasing infertility-related complications in affected women.

By enabling earlier detection, miRNAs-based diagnostics and therapies could reduce the need for invasive reproductive techniques. Furthermore, enhancing progesterone resistance displays the potential to reduce the risk of early miscarriages, thereby increasing the success rates of assisted reproductive techniques such as in vitro fertilization and intracytoplasmic sperm injection, when live birth is considered the primary outcome. Furthermore, enhanced progesterone responsiveness could improve the success rates of intrauterine insemination or even more conservative approaches, such as ovulation tracking, offering broader fertility treatment options for patients with endometriosis. Besides targeting progesterone pathways, miRNAs may influence other key processes vital for successful reproduction, such as angiogenesis, immune modulation, and oxidative stress reduction within the endometrium. Future studies are expected to explore combination therapies, where miRNA-based approaches are administered alongside conventional hormonal treatments or novel agents like antioxidants and immunomodulators.

Integrating miRNA profiling into fertility treatment protocols enables the development of personalized therapy plans, facilitating the optimization of assisted reproductive technology methods based on individual molecular profiles and enhancing the overall efficacy of treatment. Furthermore, ongoing research into the role of specific miRNAs in endometrial receptivity and embryo implantation continues to inform the development of targeted therapies aimed at enhancing fertility outcomes in endometriosis patients. Recent advances in single-cell RNA sequencing have allowed for an unprecedented understanding of miRNA expression patterns at the cellular level within the endometrium. These techniques could soon enable even more precise interventions by identifying and correcting molecular defects specific to particular endometrial cell populations.

As our understanding of the function of miRNAs deepens, it is likely that novel diagnostic markers and therapeutic targets will emerge, offering new hope for the development of personalized and effective treatment strategies in the near future. Ultimately, the integration of miRNA-based diagnostics and therapeutics holds the promise not only for improved management of endometriosis-associated infertility but also for a broader application in general reproductive medicine, potentially benefiting a wide range of patients facing challenges in conception.

## 7. Conclusions

In summary, the role of miRNAs in the pathophysiology of endometriosis and its associated infertility presents a promising avenue for both diagnostic and therapeutic advancements. The evidence discussed suggests that alterations in specific miRNAs contribute significantly to processes such as cell proliferation, migration, and hormonal regulation, which underly the development of endometriotic lesions and related fertility challenges. However, more research, especially concerning the relationship between endometriosis and infertility, is highly desirable to further elucidate these complex mechanisms. A better understanding of the interconnected pathways in this multifaceted disease could pave the way for the development of non-invasive diagnostic tools and targeted therapies. There is a hopeful prospect that continued investigations will eventually lead to improved diagnostic accuracy and more effective treatment options, ultimately enhancing fertility outcomes and quality of life for affected women.

## Figures and Tables

**Table 1:** miRNAs identified in endometriosis-associated infertility and their expression and function in this context.

miRNA	Function in Endometriosis-Associated Infertility	Expression in Endometriosis
miR-100-5p [25]	Ovarian reserve, endometrial function, endometrial receptivity	upregulated
miR-126 [17]	Unknown	upregulated
miR-135a [27]	Hormonal imbalance, implantation failure	upregulated
miR-135b [27]	Hormonal imbalance, implantation failure	upregulated
miR-145 [17]	Embryo implantation	upregulated
miR-16 [31]	Inflammatory environment	downregulated
miR-1910-3p [18]	Organ development, epithelial homeostasis, immunomodulation, fibrosis, endometrial receptivity	downregulated
miR-194-3p [31]	Hormonal imbalance, progesterone resistance	upregulated
miR-199a [31]	Inflammatory environment	downregulated
miR-21-5p [25]	Inflammatory environment	-
miR-22-5p [30]	Progesterone resistance, implantation failure	downregulated
miR-23a [31]	Hormonal imbalance	-
miR-23b [31]	Hormonal imbalance	-
miR-29c [27]	Hormonal imbalance, progesterone resistance	upregulated
miR-302d-3p [18]	Angiogenesis, proliferation of endometriosis	-
miR-31 [18]	Endometrial receptivity	downregulated
miR-3119 [18]	Unknown	-
miR-370-3p [31]	Hormonal imbalance	downregulated
miR-373-3p [18]	Inflammatory environment	-
miR-4423-3p [18]	Infertility	-
miR-4461 [18]	Unknown	-
miR-498 [18]	Inflammatory environment, decidualization of endometrial stromal cells	-
miR-501-5p [18]	Unknown	-
miR-5187-3p [18]	Unknown	-
miR-543 [29]	Implantation failure	downregulated
miR-548av-5p/548k [18]	Unknown	-
miR-548bb-3p [31]	Unknown	-
miR-6502-5p [18]	Unknown	-
miR-6503-5p [18]	Unknown	-
miR-6778-3p [18]	Unknown	-
miR-6815-3p [31]	Unknown	-
miR-6818-5p [18]	Oocyte maturation, fertilization	-
miR-6885-3p [18]	Unknown	-
miR-6893-5p [18]	Unknown	-
miR-885-5p [18]	Unknown	-
miR-9-5p [18]	Unknown	upregulated
let-7b [28]	Endometrial function	downregulated

## Data Availability

Data sharing is not applicable.

## Abbreviations

The following abbreviations are used in this manuscript:

miRNA	Micro-RNA
RNA	Ribonucleic acid
DNA	Deoxyribonucleic acid
HNF4A	Hepatocyte nuclear factor 4 alpha
AMPK	AMP-activated protein kinase
HDAC4	Histone deacetylase 4
ITGB3	Integrin beta-3 subunit
HOXA10	Homeobox A10
HOXA1	Homeobox A1
IGF1	Insulin-like growth factor 1
FKBP4	FK506-binding protein 4
TET2	Tet methylcytosine dioxygenase 2
ESR2	Estrogen receptor 2
AUC	Area under the curve
